# Application of the Forceps

**Published:** 1875-01

**Authors:** 


### APPLICATION OF THE FORCEPS.

Dr. E. H. M. Sell, in *The Physician and Pharmacist*, gives the following rules as obtaining at Vienna in the use of the forceps in obstetrical practice :

In the application of the forceps, the following three conditions are noticed as essential in the operation—

1. The cervix must be fully dilated and the head through the os and at the floor of the pelvis.

2. The forceps may be applied when the head is found in the vagina, not enveloped by the os uteri, whether it is rotated or not.

In the latter condition the blades should often be opened a little, so as to allow the head to rotate, though it frequently does so with the forceps.

3. In all cases of application of the forceps, the bladder of the woman should first be emptied. Should this be rendered difficult, from the pressure of the head upon the bladder, dividing it into two sacs, we will generally succeed by pushing the head a little up from the pubes.

4. In cases of danger to the child, the forceps should be applied, provided the conditions permit.

There is always danger : (*a*) when meconium appears ; (*b*) when the mother is exhausted ; or eclampsia threatens. When the cervix, however, is not dilated, we must allow the child to die, and then perform craniotomy, rather than run the risk of rupturing the uterus.

We would say dilate the cervix by artificial means rather than do either.

5. When the head remains a long time in the vagina and does not advance without any apparent cause.

In the latter part of a delivery the forceps are no traction-instrument, but simply a controller of the birth. allowing the head to come out gradually ; should it have to advanee too fast, we must lower the handles, or a rupture of the perineum will be the consequence. Should a rupture be eminent, episiotomy is performed in preference.

A rupture of the perineum is treated by the immediate application of serre-fines, which are usually removed in about thirty-six hours. In case of the rupture extending through the spincter-ani, a few simple sutures are applied.

In abnormal rotation of the head, we apply the forceps as usual, with this difference, that we do not sink the handles quite as much, and continue our first traction in a horizontal direction till the chin comes under the pubes; when we commence extraction, we raise the handles at an early period to bring the occiput over the perineum, and then by depressing them, the face is born under the pubes.

When there is a caput succedaneum we must push the hand as well as the forceps high up, for the tumor may be large.

*Application of the Forceps to a High-standing Head.*—In this condition the os uteri is not yet fully dilated, nor the cervix drawn back over the head of the child, which is freely movable, as it is not yet firmly fixed in the entrance of the pelvis.

In this application of the forceps, which is done only in cases of very urgent necessity, it is very easy for the head to move from side to side, causing the forceps readily to glide off, and may thus do great injury to the mother.

The woman should be thoroughly anæsthetized, and the forceps always applied laterally, guarding the blade with the hand instead of the two fingers, thus avoiding doing injury to the os.

In face presentation at the upper strait, the forceps are especially dangerous, for one blade rests on the calvaria and the other on the chin and trachea. This presentation is often the forerunner of craniotomy.

In forehead presentation at the upper strait, the face usually presents to one or the other acetabulum. In this presentation the forceps are only applied to satisfy the feelings of friends who may be standing by; while we appear to make considerable traction on them, we proceed to perform craniotomy.

We would recommend strong traction to be made, and would expect to be successful in some cases.—*Medical and Surgical Reporter, Philadelphia.*

---

To Prevent Glue Becoming Sour and Mouldy.—The addition of a quantity of carbolate of soda, just sufficient to give a strong smell to the glue, will accomplish the desired result.

